# Determinants of COVID-19 preventive health behaviors in Iranian adults based on extended parallel process model

**DOI:** 10.1038/s41598-023-41643-y

**Published:** 2023-09-02

**Authors:** Fatemeh Kordi-Kalaki, Tahereh Dehdari, Jamileh Abolghasemi

**Affiliations:** 1 Department of Health Education and Health Promotion, Faculty of Public Health, Iran University of Medical Sciences, Tehran, Iran; 2 Health Promotion Research Center, Iran University of Medical Sciences, Shahid Hemmat Highway, Tehran, Iran; 3 Department of Biostatistics, Faculty of Public Health, Iran University of Medical Sciences, Tehran, Iran

**Keywords:** Health care, Medical research, Risk factors

## Abstract

Performing preventive behaviors by individuals has been recognized as one of the important strategies for the prevention and control of Coronavirus Disease 2019 (COVID-19). This study aimed to assess the determinants of adopting preventive behaviors of COVID-19 in a sample of Iranian adults based on Extended Parallel Process Model (EPPM) variables. In this cross-sectional study, 300 adults from Tehran, Iran were selected using cluster sampling method from five areas of the city: north, south, west, east, and center. The participants completed a study instrument developed to assess demographic information and EPPM variables. Finally, the data were analyzed using SPSS software. The mean age of the participants was 40.11 (± 15.158) years. The results showed that 88.3% (n = 265) of the participants were in the process of danger control. Perceived self-efficacy, attitude, response efficiency, behavioral intention, and the number of hours of activity outside home were determinants of performing COVID-19 preventive behaviors among the participants. These predictors might be used to develop theory-based educational efforts based on EPPM variables due to encouraging people to adopt COVID-19 preventive behaviors. Our results suggest that because many participants were engaged in danger control, health professionals should focus on increasing perceived efficacy.

## Introduction

Coronavirus Disease 2019 (COVID-19) is caused by a new coronavirus called SARS-CoV-2. World Health Organization (WHO) first learned of this new virus on 31 December 2019, following a report of a cluster of cases of "viral pneumonia" in Wuhan, People’s Republic of China^1^.

In Iran, apparently the two first cases of deaths related to COVID-19 were reported on February 19, 2020, Iran^2^. In this country, up to June 2023, 7,612,234.00 Iranian people have been infected with the COVID-19 and 146,289.00 have died^3^.

During the COVID-19 pandemic, a series of studies focused on measuring individuals' knowledge, attitudes, and practices, followed by the implementation of a single educational initiative for all^4–6^. Although these endeavors have somewhat succeeded in enhancing people's compliance with health guidelines during the pandemic^4–6^, notable disparities exist among various groups regarding the adoption of behaviors aimed at preventing and controlling the spread of COVID-19^7,8^.

Given that other studies have shown people to be greatly fearful of the threat of COVID-19^9,10^, some researchers have suggested that in addition to awareness and attitude, variables such as fear of COVID-19 and threat appraisal of COVID-19 may also influence the adoption of behaviors to protect against COVID-19^11^. These variables may lead to varying responses to the COVID-19 outbreak among individuals. For example, Janahgiri et al. showed that 56.4% of their participants were engaged in danger control, while others were engaged in fear control. The researchers suggested that effectively controlling the COVID-19 pandemic requires targeted interventions that take into account people's risk perception^12^. It is worth noting that individuals in the danger control process are expected to have a higher rate of engaging in health behaviors than those in the fear control process^13^. These results highlight how differences in emotional experiences of the pandemic can inform intervention strategies^14^.

The Extended Parallel Process Model (EPPM) is a widely used model in the field of health communication and is concerned with how people respond to a perceived threat. According to this model, there are three possible responses to fear appeal messages including (1) danger control, (2) fear control, and (3) no response. These responses depend on the people's evaluation of perceived threat (the summation of perceived severity and perceived susceptibility) and efficacy (the summation of self-efficacy and response efficacy) presented in the message. To determine whether a person is in the danger control process or fear control process, we can subtract the participant’s total score of perceived threat from her/his total score of perceived efficacy. If the resulting number is positive, it shows that an individual is in the danger control process, and if the number obtained is negative, it shows the individual is in the fear control process. When in danger control, an individual is engaged in danger control responses, which include beliefs, attitudes, behavioral intention, and behavior in line with the message suggestions. When an individual is in the fear control process, he/she engages in a defensive mechanism including avoidance, denial, and reactance which aims at decreasing fear rather than performing protective behaviors to lessen the threat^15–17^.

The framework of EPPM has been used in several studies around the world to understand factors related to COVID-19 prevention behaviors. For example, Lithopoulos et al. conducted a study on a nationally representative sample of Canadians to predict physical distancing during the COVID-19 epidemic using EPPM framework. They observed that participants with high scores for both perceived threat and efficacy tended to practice more physical distancing^18^. Another study by Roberto and Zhou explained COVID-19 vaccine hesitancy among college students using the EPPM framework. They reported that the main reason for vaccine hesitancy was linked to participants’ response efficacy^19^. In another study, EPPM variables were applied to identify the factors that contributed to healthcare workers' reluctance to work during the peak of COVID-19 in Western Ethiopia. The study revealed that participants who perceived higher threat and lower efficacy were more likely to express unwillingness to continue working during the pandemic's peak^20^. Serpas et al. showed that participants’ perceived efficacy regarding preventive behaviors of COVID-19 moderated the relationship between fear and adopting preventive behaviors in young adult undergraduates^21^.

Limited research has been done in Iran on identification of the significant determinants of preventive health behaviors related to COVID-19 based on theory-based conceptual frameworks such as EPPM. However, if, based on a theoretical model such as EPPM, it is determined which control process the majority of people are engaged in and which factors play the most role in their behavior, health provider can design more appropriate and effective messages and interventions^12^. In a research conducted in Iran by Shirahmadi et al., it was discovered that the inclination of oral healthcare providers to adhere to recommended health practices in relation to COVID-19 was affected by their perceived levels of threat and efficacy^22^. Another study on how the general public perceives the COVID-19 outbreak using the variables of EPPM showed substantial variations in efficacy, defensive responses, and perceived threat among different demographics, particularly among those aged 60 and above. While women scored considerably higher than men on aspects such as self-efficacy, reactance, and avoidance, the men had higher perceived susceptibility scores compared to women^12^.

Given the prevalence of COVID-19 in Iran^3^, greater fear of COVID-19 and lower preventive COVID-19 infection behaviors among such general population groups in the country^23^ and the importance of understanding the full range of determinants of preventive measures for the COVID-19 pandemic, the present study was carried out. The objective of the study was to determine the predictors of the adoption of COVID-19 preventive health behaviors based on EPPM variables in a sample of Iranian adults.

## Methods

### Design and participants

This cross-sectional (descriptive-analytical type) study was undertaken in Tehran, Iran from October 2021 to May 2022. First, a list of the main squares in five areas (center, east, west, south, and north) of Tehran city was prepared. Due to the different socio-economic status of citizens of these five areas, one of the main squares was selected from each area by simple random sampling method.

The sample size was calculated using the following formula.

$$n = \frac{{(Z_{1 - \alpha /2} + Z_{1 - \beta } )^{2} }}{{C^{2} }} + 3,\quad C = \frac{1}{2}\ln \frac{1 + r}{{1 - r}},\quad n = \frac{{(1.96 + 0.84)^{2} }}{{0.203^{2} }} + 3 \sim 194,\quad C = \frac{1}{2}\ln \frac{1 + 0.2}{{1 - 0.2}} \sim 0.203$$

The formula utilized a confidence level of 0.95 (1 − *α*), a test power of 0.80 (1 − *β*), and a correlation value of 0.2 (*r*), resulting in the calculation of a sample size of 194. However, to account for clustering sampling with a clustering effect of 1.5, the final sample size was increased to 291. In total, 300 adult citizens who had passed by these five squares were selected. Sampling was done throughout the week and at different times of the day. In this study, the inclusion criteria were willingness to participate in the present study, age 18 years and older, and permanent residence in Tehran for more than 5 years. The present study was done according to the guidelines laid down in the Declaration of Helsinki and all procedures involving human subjects were approved by the Ethic Committee of the Iran University of Medical Sciences (no. IR.IUMS.REC.1400.635). The participants were informed about the objectives of the study and completed a written consent form.

### Data gathering

In this study, a researcher-made instrument was developed by the research team and used to identify EPPM variables. Based on the literature review about EPPM and preventive behaviors of COVID-19, initial items of the instrument were designed. In this step, a 52-item instrument with 9 subscales including response efficacy (7 items), perceived self-efficacy (8 items), perceived susceptibility (4 items), defense mechanisms (9 items), perceived severity (6 items), behavioral intention (3 items), fear (3 items), attitude (4 items), and preventive behaviors of COVID-19 (8 items) was developed. In the next step, the validity of the developed instrument was measured. To measure the quantitative and qualitative content validity of the instrument items, a panel of 7 experts in health education and infectious disease specialist was used. Based on their opinion, Content Validity Index (CVI) and Content Validity Ratio (CVR) of each item were assessed. In addition, we considered their comments in terms of grammar, wording errors, usage of appropriate words (qualitative content analysis). The CVR has been defined as direct linear transformation of a proportional level of agreement on the number of experts evaluating an item as essential. The CVR formula is as follows: CVR = (ne − N/2)/(N/2), where CVR is the content validity ratio, ne is the number of panelists which evaluate an item as “essential”, and N is the total number of panel members. The necessity of the items was assessed using a three-point rating scale as essential (3), useful but not essential (2), and not necessary (1). The CVI is also measured by counting the number of experts who identified the item as 3 or 4 and dividing it by their total number. Moreover, the relevance of the items was assessed through a four-point rating scale as follows: not relevant (1), slightly relevant (2), relevant (3), and very relevant (4)^24,25^. At this step, 2 items (1 item of self-efficacy belief variable and 1 item of perceived severity variable) with CVI < 0.79 and CVR < 0.99^24,25^ were deleted and 9 items were also edited. At the end of this step, the experts approved the instrument items. In this study, the face validity of the instrument items was measured qualitatively and quantitatively. Ten adults with similar characteristics to the target population of the study judged on the relevance, ambiguity, and difficulty of the instrument items. Based on their comments on the importance of the items, we measured the impact score of each item and edited some wording errors. Impact score ≥ 1.5 was considered as acceptable^26^. All items had an impact score of ≥ 1.5 and no item was deleted (Table 1).

**Table 1:** Factor, factor loading, content validity index, content validity ratio and impact score of the instrument items developed (n = 300).

Items	Factor									CVI	CVR	Impact score
	1	2	3	4	5	6	7	8	9			
Wearing a mask may prevent getting infected with COVID-19	0.888									1	1	4.7
Washing your hands regularly with soap and water or disinfecting them can prevent infection with COVID-19	0.833									1	1	4.6
If I do the necessary health actions to prevent COVID-19, I will be less likely to get this disease	0.828									1	1	4.7
If all members of the society do not leave the house unnecessarily, the transmission cycle of COVID-19 virus will be interrupted	0.783									1	1	4.4
Maintaining a social distance (1.5–2 m) with others is effective in the prevention of COVID-19	0.770									1	1	4.6
Not participating in parties and ceremonies is an effective behavior in the prevention of COVID-19	0.761									1	1	4.8
Establishing proper ventilation in closed environments is an effective method in the prevention of COVID-19	0.742									1	1	4.7
I can do health behaviors to prevent COVID-19, wherever I am		0.808								1	1	4.4
I can tolerate the mask on my face		0.548								1	1	4.5
I can wash my hands with soap and water frequently, even though it is time-consuming		0.846								1	1	4.4
Even though I'm bored, I can avoid leaving the house when it's not necessary		0.852								1	1	4.5
I can maintain a suitable social distance with others (1.5–2 m) outdoors		0.869								1	1	3.9
I can say no to others to attend parties and events		0.772								1	1	4.3
I can create proper ventilation in closed environments around me even though the weather is cold		0.548								1	1	4.4
I am not at risk of infection with COVID-19			0.931							1	1	4.1
It is not possible that I will be infected with COVID-19			0.894							1	1	4.7
Only elderly people get infected with COVID-19			0.871							1	1	4.3
COVID-19 is a serious and dangerous threat due to the high speed of virus transmission				0.883						1	1	4.7
COVID-19 virus can affect the lungs				0.828						1	1	4.7
Covid-19 can be fatal				0.776						1	1	4.7
During the peak of the disease, it is difficult to access treatment for Covid-19				0.745						1	1	4.6
Covid-19 threatens the individuals’ mental health				0.662						1	1	4.6
I am willing to do health behaviors to prevent Covid-19					0.954					1	1	4.5
I have planned to do health behaviors to prevent infection with COVID-19					0.938					1	1	4.1
I intend to do health behaviors to prevent COVID-19					0.926					1	1	4.4
The messages and information about COVID-19 make me feel scared						0.967				1	1	4.5
For me, the messages and information about COVID-19 are scary						0.948				1	1	4.4
The messages and information about COVID-19 worry me						0.942				1	1	4.2
Performing health behaviors to prevent COVID-19 is good/bad							0.950			1	1	4.2
Performing health behaviors to prevent COVID-19 is effective/ineffective							0.916			1	1	4.2
Performing health behaviors to prevent COVID-19 is important/ unimportant							0.873			1	1	4.1
Performing health behaviors to prevent COVID-19 is useful/ worthless							0.853			1	1	4.3
I wash my hands several times a day with soap and water, and if soap and water are not available, I use alcohol								0.852		1	1	4.5
When I leave the house, I put on a mask								0.849		1	1	4.5
I don't leave my home except when necessary								0.836		1	1	4.9
I maintain a social distance (1.5–2 m) outside the house and in contact with others								0.826		1	1	4.3
I avoid participating in events and parties								0.764		1	1	4.6
I go to medical centers when I experience the symptoms of COVID-19 (such as fever and cough)								0.752		1	1	4.8
I listen to information and recommendations of health professionals about COVID-19								0.688		1	1	4.9
I try to create a proper ventilation in closed environments								0.619		1	1	4.5
The news and information presented about COVID-19 are trying to put pressure on me in their own way									0.948	1	1	4.2
Those who write about COVID-19 want to take control of me									0.947	0.85	1	3.2
COVID-19 is a misleading story									0.939	0.85	1	3.8
COVID-19 has been manipulative									0.926	1	1	4.1
I don't listen when information about COVID-19 is presented									0.902	1	1	3.7
I don't want to read about COVID-19									0.900	1	1	3.6
Information about COVID-19 has been overstated									0.895	1	1	3.9
COVID-19 has been overblown									0.889	1	1	3.9
The severity of COVID-19 has been exaggerated									0.888	1	1	3.9

In the next step, Exploratory Factor Analysis (EFA) was conducted. In the study, the EFA samples were identical to the study samples, and no additional sampling was conducted. Literature showed that a minimum of 300–450 was needed to observe an acceptable comparability of patterns^27^. In the study, for performing EFA, 300 adults filled out the instrument. Data collected in this stage were analyzed using SPSS software. The Kaiser–Meyer–Olkin (KMO) test and the Bartlett’s test of sphericity of each subscale were used to assess the adequacy of the sample and appropriateness of the factor analysis model. The final items in each subscale which were included in the proposed research model was selected based on the communality indexes above the threshold of 0.4; also, the latent root criterion of 1.0 was used for factor extraction^28,29^. The KMO test expectancies of the instrument was acceptable (Table 2)^30^. The results of this step showed that Bartlett’s test of sphericity of the instrument was significant. Also, Eigen values of the instrument were > 1. The factor loads and the total variance of the instrument is shown in the Tables 1 and 2^31^. In this stage, no item was deleted from the subscales. Finally, to measure the reliability of the stability, we used the test–retest correlation coefficients (with a 2-week interval between the tests) on thirty adults with similar characteristics to the target population of the study. In this step, Infraclass Correlation Coefficient (ICC) was calculated. ICC ≥ 0.70 was considered satisfactory^30^. Based on the data obtained from completing the instrument by 30 other adults, we assessed Cronbach's alpha^30,32^ to find the internal consistency of the items of each subscale of EPPM. Cronbach's alpha ≥ 0.70 was considered acceptable^30,32^. In this step, one item was removed from the perceived susceptibility subscale items. ICC and Cronbach's alpha of EPPM variables in terms of adopting COVID-19 preventive behaviors were shown in Table 3. The final 49-item instrument had 9 subscales (7 items for response efficacy, 7 for perceived self-efficacy, 3 for perceived susceptibility, 9 for defense mechanisms, 5 for perceived severity, 3 for behavioral intention, 3 for fear, 4 for attitude and 8 for preventive behaviors of COVID-19). The items of response efficacy, perceived self-efficacy, perceived severity, and behavioral intention were measured with a 5-point Likert scale (“completely agree” = 5 to “completely disagree” = 1). The items of perceived susceptibility and defense mechanisms were measured with a 5-point Likert scale (“completely disagree” = 5 to “completely agree” = 1). Fear was measured using a 7-point Likert scale (“completely agree” = 7 to “completely disagree” = 1). Preventive behaviors of COVID-19 were also measured on a 5-point scale (“always” = 5 to “never” = 1). Four items were employed to measure the attitude (for example: “adopting COVID-19 preventive behaviors is pleasant for me”). These items were measured on a 7-point Likert scale ranging from 7 (completely disagree) to 1 (completely agree). The study researchers developed a questionnaire to identify demographic characteristics of the participants, such as their age, gender, marital status, education level, occupation, and the number of hours they spend on activities outside of their home. With the consent of the participants, a more private place (such as a bench on the sidewalk) was selected to complete the instruments. The duration of completing the instrument by each participant was about 15 min.

**Table 2:** The results of explanatory factor analysis.

Factors	Factor label	Eigenvalue	Cumulative variance (%)	Kaiser–Meyer–Olkin measure of sampling adequacy	Approx. Chi-square	df	Sig
1	Response efficacy	1.093	75.431	0.925	13,474.889	1176	< 0.001
2	Self-efficacy						
3	Perceived susceptibility						
4	Perceived severity						
5	Behavioral intention						
6	Fear						
7	Attitude						
7	Behavior						
8	Defense mechanisms

**Table 3:** Intraclass correlation coefficient (ICC) and Cronbach's alpha of extended parallel process model variables (n = 300) in terms of adopting COVID-19 preventive behaviors.

Variables	The number of items	ICC	Cronbach's alpha
Response efficacy	7	0.996	0.945
Perceived self-efficacy	7	0.998	0.929
Fear	3	0.997	0.977
Perceived severity	5	0.998	0.838
Perceived susceptibility	3	0.885	0.700
Defense mechanisms	9	0.994	0.962
Attitude	4	0.997	0.949
Behavioral intention	3	0.993	0.962
Adopting COVID-19 preventive behaviors	8	0.989	0.918

### Statistical analysis

The data were analyzed using SPSS software package (version 18.0, SPSS, Inc., Chicago, IL, USA). Normality of data was tested using Kolmogorov–Smirnov test. Pearson's correlation test was used to check the correlation between EPPM variables. Multiple linear regression was used to evaluate the factors effective on adopting preventive behaviors as to COVID-19. The participants’ general characteristics were analyzed using frequency, percentage, mean and standard deviation. In the present study, the level of significance was considered p < 0.05. All the participants read a statement that explained the purpose of the study and provided written informed consent before participation in the study. The participants were reminded that the study was voluntary, confidential and the results would remain anonymous.

### Consent to participate

All the participants read a statement that explained the purpose of the study and provided written informed consent before participation in the study. The participants were reminded that the study was voluntary, confidential and the results would remain anonymous. Ethics approval was obtained from the Ethics Committee of Iran University of Medical Sciences (no. IR.IUMS.REC.1400.635).

## Results

The mean age of the participants was 40.11 (SD = 15.158) years. Table 4 shows other demographic characteristics of the participants. The result showed that there was a significant correlation between the mean score of adopting COVID-19 preventive behaviors and all EPPM variables (except for perceived threat) (Table 5).

**Table 4:** Demographic characteristics of the study participants and the relationship between them with the score of adopting COVID-19 preventive behaviors (n = 300).

Variables	Mean ± SD	n%	*p* value
Age	40.11 ± 15.158		0.029
Gender			
Female		171 (57)	0.53
Male		129 (43)	
Marital status			
Single		113 (37.7)	0.567
Married		187 (62.3)	
Educational level			
Elementary		26 (8.7)	< 0.001
Middle school		33 (11)	
High school		34 (11.3)	
Diploma		74 (24.7)	
University		133 (44.3)	
Occupation			
University student		35 (11.7)	0.024
Employee		59 (19.7)	
Self-employed		60 (20)	
Casual labourer		37 (12.4)	
Household duties		109 (36.2)	
The number of hours of activity outside home	6.64 ± 4.581		0.083

Significance at the 0.2 level.

**Table 5:** Correlation between extended parallel process model variables (n = 300).

Variables	Response efficacy	Perceived susceptibility	Defense mechanisms	Perceived severity	Behavioral intention	Fear	Preventive behaviors	Self-efficacy	Attitude	Perceived threat	Perceived efficacy
Response efficacy	–										
Perceived susceptibility	− 0.145*	–									
Defense mechanisms	0.295**	0.611**	–								
Perceived severity	0.369**	− 0.033	0.175**	–							
Behavioral intention	0.680**	− 0.182**	0.218**	0.400**	–						
Fear	0.329**	− 0.102	0.070	0.317**	0.292**	–					
Preventive behaviors	0.739**	− 0.214**	0.168**	0.307**	0.721**	0.322**	–				
Self-efficacy	0.823**	− 0.199**	0.189**	0.279**	0.676**	0.313**	0.825**	–			
Attitude	0.501**	0.029	0.215**	0.284**	0.467**	0.336**	0.525**	0.476**	–		
Perceived threat	0.114	0.765**	0.595**	0.599**	0.110	0.126*	0.033	0.020	0.200**	–	
Perceived efficacy	0.948**	− 0.178**	0.251**	0.329**	0.712**	0.334**	0.821**	0.961**	0.513**	0.064	–

*Significance at 0.05 level; **significance at 0.01 level.

Of the participants, 88.3% (n = 265) engaged in danger control while 11.7% (n = 35) engaged in fear control. Given that the results of single linear regression showed that age, educational level, occupation status, the number of hours of activity outside home, history of COVID-19 in participants and first-degree relatives, status of hospitalization of the participants and first-degree relatives with COVID-19, and number of vaccine injections and EPPM variables were significant at the level of 0.2, these variables were candidate for multiple linear regression. Table 6 shows the results of multiple linear regression of the estimation model of COVID-19 preventive behaviors based on EPPM variables and demographic information in the study participants. Findings showed that perceived self-efficacy, attitude, response efficacy, behavioral intention, and the number of hours of activity outside home were identified as significant determinants preventive measures for the COVID-19 pandemic among the participants (Table 6). As shown in this Table, by increasing one unit in the number of hours of activity outside home, behavioral intention, attitude and perceived self-efficacy variables, adopting the COVID-19 preventive behavior increased by 0.144, 0.423, 0.229, and 0.605, respectively. In addition, by increasing one unit in the response efficiency variable, the target behavior decreased by 0.481 (Table 6).

**Table 6:** The results of the multiple linear regression model for the estimation of COVID-19 preventive behaviors based on extended parallel process model variables in the study participants (n = 300).

Variables	Coefficient	SD	Beta	p value
Constant	− 0.706	2.023	–	0.728
The number of hours of activity outside home	0.144	0.087	1.121	< 0.001
Behavioral intention	0.423	0.099	0.117	0.022
Response efficacy	− 0.481	0.172	− 0.443	0.006
Attitude	0.229	0.165	0.166	0.012
Self-efficacy	0.605	0.068	0.086	0.035

Significant, p < 0.05.

## Discussion

In this study, we observed a larger number of participants (88.3%) engaging in the fear control process compared to some previous studies. For example, Jahangiry et al. reported that only 56.4% of their participants in Iran engaged in danger control process, while others engaged in fear control process^12^. The difference in results may be attributed to the timing of the studies. Previous studies were conducted during the early stages of the COVID-19 outbreak when fear and anxiety surrounding the infection were high in the community^9^. However, as time has passed and more people have been vaccinated, it is possible that the percentage of individuals engaging in danger control behaviors has increased. While stress caused by the fear of COVID-19 may motivate some individuals to engage in preventive behaviors, psychological distress caused by this fear can also hinder preventive health behavior^33^.

The results showed that among the components of efficacy appraisal (including response efficacy and perceived self-efficacy), self-efficacy had a direct positive effect on performing recommended COVID-19 preventive behaviors among the participants. Greater self-efficacy belief resulted in an increase in performing COVID-19 preventive behaviors in them. In many studies, self-efficacy has been recognized as the strongest predictor of adopting healthy behaviors^34,35^. Similar to our study, Khazei et al., Fang et al., and Mahmood et al. concluded that the individuals’ self-efficacy should be improved in order to prevent COVID-19^36–38^. Motayerzadeh et al. found that in order to increase COVID-19 preventive behaviors, we need to consider and increase the individuals’ self-efficacy^39^. In this study, self-efficacy and response efficacy had positive relationships with EPPM variables (except for perceived susceptibility). In other words, as perceived sensitivity increased, self-efficacy and response efficacy decreased. The reason for this may be that when people do not perceive themselves to be susceptible to contracting COVID-19, they do not feel the need to perform preventive behaviors and often have no feelings about their ability to perform the recommended behaviors and the behaviors are not considered very effective. Given that the self-efficacy is one of the mediating factors between gaining knowledge through exposure to multiple communication channels and performance of preventive behaviors and has a strong effect on danger control process of COVID-19^40^, it is necessary to further emphasize this variable in designing the interventions and education health messages. Using vicarious experiences, improving the physiological state, encouraging the individuals to repeat the recommended behavior, simplifying the recommended behavior and verbal persuasion may help to increase the individuals’ self-efficacy belief to adopt a healthy behavior^41^.

Contrary to our expectations, the effect of perceived response efficacy on the behavior was significant and inverse. In other words, the participants who perceived higher levels of response efficacy adopted the COVID-19 preventive behaviors less frequently than the participants who perceived less response efficacy. The reason for this finding may be that people have often observed and heard from others that despite taking preventative measures, they still get infected with COVID-19. In a study, Roberto and Zhou showed that the main reasons for doubting the injection of the COVID-19 vaccine were safety and efficacy of the vaccine in the prevention of COVID-19^19^. The finding of the present study was inconsistent with these previous studies. For example, Khazaei et al. reported that individuals' beliefs about the effectiveness of recommended behaviors were important in preventing COVID-19^36^. Motayerzadeh et al. also indicated that increasing the acceptance of the COVID-19 vaccine should be emphasized by highlighting the effectiveness of the vaccine^39^. The result of a study by Koebele et al. suggested that messaging focused on the relative ease and effectiveness of mask wearing (response efficacy) may help increase compliance with public health recommendations for mitigating COVID-19^42^. The reason for the difference between the findings of the current study and other studies may be the different populations that were studied and the normalization of living with COVID-19 by people after several years of its epidemic in Iran.

The results of the present study showed that by increasing one unit in behavioral intention, adopting the COVID-19 preventive behaviors by the participants increased by 0.423. Literature showed that various factors may impact on the individuals’ intention for adopting a specific behavior^19,43^. For example, Tsoy et al. also found that factors such as efficacy and threat had positive effects on the intention of people to stay at home during the COVID-19 pandemic^43^. Also, the results of Roberto and Zhou's study showed that the intention to receive COVID-19 vaccine significantly predicted COVID-19 vaccine injection behavior^19^.

In the study, there was a significant relationship between intention and all variables of EPPM (except for perceived threat). In contrast to our study, Tsoy et al. reported that the intention to stay at home during the COVID-19 pandemic was considerably high when the threat was high^43^. The reason for this different finding is that only 11% of participants in the present study were in the process of fear control, while most of the participants were in the danger control process. Based on EPPM assumptions, if a person is engaged in the danger control process, their score of perceived efficacy in terms of a threatened condition should be higher than their score of perceived threat^41^. Therefore, to develop effective education measures, it is necessary to focus on the significant factors influencing behavioral intention in both the danger and fear control processes.

The results of the study showed that an increase of one unit in the attitude variable, the score of performing the COVID-19 preventive behaviors increased by 0.229. Consistent with our study, Hong et al. reported that believing in the safety of the vaccine, willingness to pay for it, and consent to recommend families and friends to get vaccinated were the contributors to COVID-19 vaccine acceptance among patients with cancer^44^. In other study, Kim et al. concluded that belief about COVID-19 had a significant positive effect on the practice of health college students in terms of prevention behaviors of COVID-19^45^. In this study, there were significant correlations between attitude and all variables of EPPM (except for perceived susceptibility). This finding is consistent with such studies in the field of COVID-19. For example, Rakotoarisoa et al. demonstrated that attitude had a strong positive relationship with intention behavior to performing preventive behaviors against COVID-19^46^. Given that modifying attitude towards a specific behavior may lead to changes in the behavior^47^, it is necessary to identify the people's attitudes on COVID-19 and the preventability of the disease by adopting healthy behaviors and try to change unfavorable attitudes through the use of interactive educational methods such as group discussion, role playing, and peer group.

The number of hours of activity outside home was one of the significant determinants of performing the COVID-19 preventive behaviors among our participants. Probably, people who spend more hours outside home in public places have greater perceived threat on COVID-19. Therefore, these people performed COVID-19 preventive behaviors more frequently than other people. In line with this finding, Zare et al. reported that the level of outdoor sports activity may be considered as one of the main predictors of performing behaviors effective on prevention of COVID-19^48^. Also, the finding of the present study was inconsistent with findings of a study by Ghiasvand and Taghizadeh. They showed that more people had to leave home because of their occupation. They less frequently performed COVID-19 preventive behaviors^49^. It is necessary to pay more attention to the education of people who spend more hours of the day outside home (e.g. employee) in terms of the advantages of performing COVID-19 preventive behaviors.

In the present study, the components of threat appraisal including perceived susceptibility and perceived severity had not significant impacts on the behavior. The reason for this finding is that most of the participants studied (about 90%) were engaged in danger control process. Literature showed that the participants in the danger control process were more likely to take protective action to avoid or reduce the threat of COVID-19, while those in the fear control category did not feel at risk and were unlikely to take preventive measures^50^. This finding of the present study was inconsistent with such similar previous studies. For example, Shirahmadi et al. concluded that the dominance of perceived threat weight over perceived efficacy may lead to increasing practice of recommended COVID-19 preventive behaviors^13^. Saadat et al. also found that risk perception was the only significant predictive variable in terms of performing COVID-19 preventive behaviors^51^. Such research has shown that designing messages with a balance between perceived threat and perceived efficacy can be more effective in encouraging people to adopt the recommended COVID-19 preventive behavior^19^. Tsoy et al. also concluded that people were likely to stay at home during COVID-19, as a preventive measure, if there were sufficient threats and efficacy in them^43^. Our results indicated that because many study participants were engaged in danger control, so health professionals should focus on increasing the efficacy appraisal components. Risk communication during COVID-19 outbreak should provide customized information on people who do not believe in the efficacy of the recommended health behaviors in the prevention of a specific disease. Besides delivering the message with a focus on perceived self-efficacy, health professionals or mass media should make an attempt to create a social environment in which people encourage each other to adopt the recommended preventive behaviors effectively and perform those behaviors better.

Although this study highlights the significant determinants of adopting COVID-19 preventive behaviors based on EPPM variables in a sample of adults in Tehran, Iran, it has some limitations. The first limitation of the study was due to data collection problems during the COVID-19 epidemic, which resulted in identical EFA samples and no additional sampling. The second limitation was that the study was conducted solely in the city of Tehran and limited to a sample of citizens in the urban area. This was due to the short timeframe and limited resources available for conducting the study in a single area. Therefore, we recommend expanding this study to other geographical areas in Iran.

## Conclusion

Five variables including perceived self-efficacy, attitude, response efficiency, behavioral intention, and the number of hours of activity outside home were recognized as were identified as significant determinants of adopting the COVID-19 preventive behaviors among the participants. These variables may be used as focal points for educating people on how to reduce their risk of exposure to COVID-19 in the urban areas of Tehran, Iran.

## Data Availability

The datasets used and/or analysed during the current study are available from the corresponding author on reasonable request. Please contact the corresponding author for the data requests.
